# On-Field Test of Tuberculosis Diagnosis through Exhaled Breath Analysis with a Gas Sensor Array

**DOI:** 10.3390/bios13050570

**Published:** 2023-05-22

**Authors:** Yolande Christelle Ketchanji Mougang, Laurent-Mireille Endale Mangamba, Rosamaria Capuano, Fausto Ciccacci, Alexandro Catini, Roberto Paolesse, Hugo Bertrand Mbatchou Ngahane, Leonardo Palombi, Corrado Di Natale

**Affiliations:** 1 Department of Electronic Engineering, University of Rome Tor Vergata, via del Politecnico 1, 00133 Roma, Italy; ketchanji.mougang@ing.uniroma2.it (Y.C.K.M.); rosamaria.capuano@gmail.com (R.C.); catini@ing.uniroma2.it (A.C.); 2 Faculty of Medicine and Pharmaceutical Sciences, University of Douala, Carrefour Ange Raphael, Douala P.O. Box 4035, Cameroon; endalem@yahoo.fr (L.-M.E.M.); mbatchou.ngahane@yahoo.com (H.B.M.N.); 3 Center for Respiratory Diseases, Douala Laquintinie Hospital, Avenue du Jamot, Douala P.O. Box 4035, Cameroon; 4 Interdepartmental Centre for Volatilomics “A D’Amico”, University of Rome Tor Vergata, via del Politecnico 1, 00133 Roma, Italy; paolesse@uniroma2.it; 5 UniCamillus, Saint Camillus International University of Health and Medical Sciences, 00131 Rome, Italy; fausto.ciccacci@unicamillus.org; 6 Department of Chemical Science and Technology, University of Rome Tor Vergata, via della Ricerca Scientifica, 00133 Rome, Italy; 7 Internal Medicine Service, Douala General Hospital, Douala P.O. Box 4856, Cameroon; 8 Department of Biomedicine and Prevention, University of Rome “Tor Vergata”, Viale Montpellier 1, 00133 Roma, Italy

**Keywords:** tuberculosis (TB), breath analysis, volatile organic compounds (VOCs), electronic nose

## Abstract

Tuberculosis (TB) is among the more frequent causes of death in many countries. For pulmonary TB, early diagnosis greatly increases the efficiency of therapies. Although highly sensitive tests based on nucleic acid amplification tests (NAATs) and loop-mediated isothermal amplification (TB-LAMP) are available, smear microscopy is still the most widespread diagnostics method in most low–middle-income countries, and the true positive rate of smear microscopy is lower than 65%. Thus, there is a need to increase the performance of low-cost diagnosis. For many years, the use of sensors to analyze the exhaled volatile organic compounds (VOCs) has been proposed as a promising alternative for the diagnosis of several diseases, including tuberculosis. In this paper, the diagnostic properties of an electronic nose (EN) based on sensor technology previously used to identify tuberculosis have been tested on-field in a Cameroon hospital. The EN analyzed the breath of a cohort of subjects including pulmonary TB patients (46), healthy controls (38), and TB suspects (16). Machine learning analysis of the sensor array data allows for the identification of the pulmonary TB group with respect to healthy controls with 88% accuracy, 90.8% sensitivity, 85.7% specificity, and 0.88 AUC. The model trained with TB and healthy controls maintains its performance when it is applied to symptomatic TB suspects with a negative TB-LAMP. These results encourage the investigation of electronic noses as an effective diagnostic method for future inclusion in clinical practice.

## 1. Introduction

Even though it has been well identified since the 19th century and even after a century of vaccination, tuberculosis (TB) is still the world’s leading cause of infectious death [1]. According to the Global Tuberculosis Report of the World Health Organization, in 2021, 7.8 million people suffered from active TB, and about 1.4 million died from the disease [2]. Over 95% of TB deaths occur in low- and middle-income countries, and within these countries, the prevalence of TB is higher among the impoverished [3].

Nevertheless, TB is preventable and treatable. The observation of the basic patterns of clinical symptoms (4SS: fever, cough lasting more than 2 weeks, night sweats, loss of weight or appetite) complemented by chest X-ray provides the first step for a diagnosis, which is typically confirmed using microbial culture-based tests, which require a long incubation of the sputum sample. As an alternative, a smear microscopy (SMEAR) test can provide a result in a few hours but with a sensitivity lower than 65% [4]. Due to its moderate cost, SMEAR is the current standard point-of-care (POC) test in low- and middle-income countries [5,6].

Important advances in TB diagnosis are offered by molecular tests that produce reliable nucleic acid amplification tests (NAATs). For example, the introduction of GeneXpert (Xpert MTB/RIF) and line probe assays have significantly increased the sensitivity and shortened the time for a TB diagnosis with a sensitivity of 97–99% for smear-positivity [7]. The introduction of NAATs is expected to greatly improve the containment of TB. However, NAATs still require sputum collection, trained staff, and two hours for the response. Additional methods aimed at microorganisms’ detection, such as the TB-LAMP (loop-mediated isothermal amplification) can provide results in less than one hour, with sensitivity and specificity for smear- and culture-positivity of 90% and 95% [8]. However, both these methods are more expensive than SMEAR [9].

In this context, there is an urgent need to develop rapid, low-cost, and easy-to-use methods for TB diagnosis that can ensure effective screening in the field, the rapid isolation of infected subjects, and the control of the efficiency of therapies. In this regard, the measure of the volatile fraction of the metabolome, called the volatilome, has been proposed as a viable alternative to the current diagnostics methods. Many studies present evidence that a vast range of phenomena, both in vitro and in vivo, are characterized by specific patterns of VOCs [10]. With respect to VOC production, breath is the richest source of volatile compounds, also in terms of chemical diversity [11], and breath composition has been found to be correlated with different pathologies and conditions [12].

The relationship between the breath volatilome and tuberculosis has been investigated in several papers using analytical instrumentations such as gas chromatography coupled with mass spectrometry (GC-MS) [13,14,15]. Most of the volatile compounds related to TB are aldehydes, methylated aromatics, alkanes, and alkane derivatives. Most of these molecules are also general products of oxidative stress [16]. It is worth noting that in TB, the changes in volatilome composition are not only due to the patient’s response to the infection but also to the metabolites of the mycobacterium itself [17].

The interest in volatilome analysis has been further increased by the introduction of miniaturized gas sensors, which are connected to microelectronics platforms to give rise to simple-to-use and low-cost devices [18,19,20]. These devices enable the design of arrays of gas sensors that can detect the composition of complex mixtures of compounds, implementing the same approach as that of natural olfaction [21]. The diagnostic properties of these devices, also known as electronic noses, have been demonstrated with different kinds of samples, such as breath [22], urine [23], sweat [24], saliva [25], and cell cultures [26]. Among them, the detection of tuberculosis has so far been attempted with electronic noses based on different sensor technologies such as metal oxide semiconductors [27,28], organically capped gold nanoparticles [29], and porphyrin-coated quartz microbalances [30].

In this paper, an electronic nose made of porphyrinoid-functionalized quartz microbalance sensors was tested in a measurement campaign at the Laquintinie Hospital in Douala (Cameroon). The study involved diagnosed TB patients, patients suspected to have TB but affected by different diseases, and a group of healthy controls. A preliminary version of this EN was previously used to identify TB in a study carried out in Botswana [30]. With respect to that version, the EN used here contained an extended array of sensors where corrole-based sensors were added to the previously used porphyrins, along with a mass flow controller that improved the reproducibility of the measurement and the monitoring of breath humidity and temperature. The current version of the EN was robust enough to be operated in a hospital outside the controlled laboratory environment.

The results are encouraging with respect to the possibility of developing a dedicated instrument that complies with the general requirements for POC, such as appropriate price, sensitivity, specificity, user friendliness, fast response, reliability, and accessibility [31].

Finally, it is important to point out the geographical relevance of this study. Indeed, tuberculosis is endemic in Cameroon. According to the World Health Organization, in 2021, this country was 3rd out of the top 20 countries in terms of the incidence of TB cases among people living with HIV [2], and TB caused about 13,100 deaths in 2017 [32].

## 2. Materials and Methods

### 2.1. Study Design and Recruitment

This study was an interventional case–control study on adults (all genders over 18 years). The study protocol follows the WHO guidelines, and it was approved by the Institutional Ethic Committee at the Faculty of Medicine, University of Douala (No 2341/IEC-UD/07/2020/T) and registered after review acceptance at ClinicalTrials.gov (NCT02219945). Participants were recruited from the Center of Respiratory Disease of the public Laquintinie Hospital in Douala (Cameroon). Signed informed consent forms were obtained from each patient before enrollment. The cohort included patients with diagnosed TB, patients with suspected TB, and healthy controls. All suspected TB patients were diagnosed using a sputum test with TB-LAMP. Other clinical analyses were collected for suspected TB patients, such as SMEAR and chest X-rays. All patients were able to expectorate sputum and exhale breath samples. Healthy controls were free of any of the 4SS of pulmonary TB, and without a respiratory disease history. After breath tests, the study participants were followed-up in time to confirm TB diagnosis. In cohort selection, people with a cancer history were excluded because of the previously mentioned effects of cancer on VOC profile. For each participant, demographic and clinical data and blood/urine laboratory tests were also collected.

Measurements were collected in a period of two months. Healthy and TB subjects were measured in a random sequence to avoid any effect of a possible drift of sensor signals.

### 2.2. Breath Sampling and Processing

**Figure 1:**
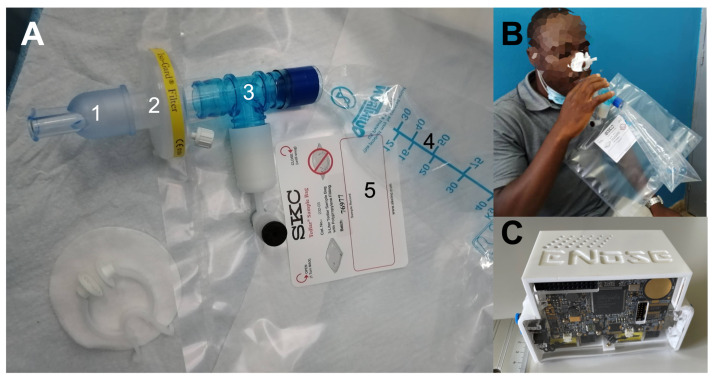
Breath sample collection. (**A**) Assembled sampler: 1: mouthpiece, 2: anti-bacterial filter, 3: 3-way valve, 4: bag for dead space collection, 5: bag for alveolar breath collection. (**B**) Example of use. (**C**) Picture of the EN unit.

A breath sampler was designed by modifying a breath sampler previously designed to separate alveolar breath from the dead space [33]. The breath sampler is shown in Figure 1. During the collection, subjects wore a nose clip and were asked to inhale their total lung capacity and to exhale deeply into a mouthpiece equipped with a bacterial and viral Gibeck ISO-Gard Filter (TeleFlex, Morrisville, NC, USA)). The breath was portioned into two bags. The access to the first bag (volume 400 mL, from QuinTron Instruments, Milwaukee, WI, USA) was always open, while the access to the second larger bag (a 3 l volume tedlar sample bag, SKC Inc., Eighty Four, PA, USA) was controlled by a three-way valve (QT00854/5-P from QuinTron Instruments, Milwaukee, WI, USA). The first portion of breath fills the small bag, and as the pressure increases, the three-way valve opens and the larger bag is filled. Such a system separates the alveolar part of the breath into the larger bag and it improves the rejection of the sources of VOCs located in the upper respiratory part. The content of alveolar breath was immediately measured on site.

All subjects were required to refrain from consuming food, drinking, or smoking for at least two hours before the breath collection. All subjects spent at least 10 min in the measurement room prior to breath testing to normalize their lungs to the environmental air.

### 2.3. Electronic Nose

The gas sensor array was an ensemble of eleven quartz microbalances (QMB). QMBs are mass sensors where the changes in the mass (Δm) at the quartz surface result in frequency changes (Δf) in the electrical output signal of an oscillator circuit to which each sensor is connected; in the low-perturbation regime, the sensitivity of the sensor is ruled by the Sauerbrey law, and Δm and Δf are linearly proportional [34].

The QMBs used in this paper had a fundamental frequency of 20 MHz (KVG Gmbh, Neckarbischofsheim, Germany), corresponding to a mass resolution of the order of a few nanograms. The sensitive molecules are listed in Table 1. They are differently functionalized forms of metalloporphyrins and corroles [35,36]. Sensors 1 to 7 are different metal complexes of tetraphenylporphyrins functionalized with a butyloxyphenyl group to ensure the diffusion of target molecules through the sensitive film. Sensors 8 and 9 are freebase and molybdenum complexes of tetraphenylprorphyrins decorated with bromium atoms. Finally, sensors 10 and 11 are corrole macrocycles functionalized with dimethylphenyl and phenantryl groups, respectively.

The QMBs were assembled in a sensor system endowed with airflow inlet control and communication with a computer. This EN has been used for human volatilome studies such as breath analysis to diagnose lung cancer [37], urine analysis for kidney cancer [38], and blood serum for COVID-19 [39]. The EN unit is shown in Figure 1C.

In each measurement, the 3 l Tedlar bag was connected to the EN inlet. The cleaning inlet of the EN was always connected to the environment through a dryer calcium chloride trap. Each measurement cycle consisted of air flowing into the bag for 180 s followed by a cleaning phase of 540 s, during which the EN was flushed with ambient air filtered by the calcium chloride trap. The response of each sensor was evaluated as the difference between the frequency measured at the end of the exposure to the breath sample and the frequency measured immediately before the exposure to the sample with the sensors exposed to filtered ambient air. For each bag, a sequence of three measures were taken. Thus, for each subject, three measurements were taken.

### 2.4. Statistical Analysis

The statistical significance of sensor signals was evaluated with the non-parametric Kruskal–Wallis rank sum test. Multivariate analysis was carried out with the principal component analysis (PCA) and linear discrimination analysis (LDA) algorithms.

PCA is a simple algorithm to explore the multivariate distribution of sensor data. It provides the projection of the multidimensional data onto a plane defined by the directions of maximum correlation.

**Figure 2:**
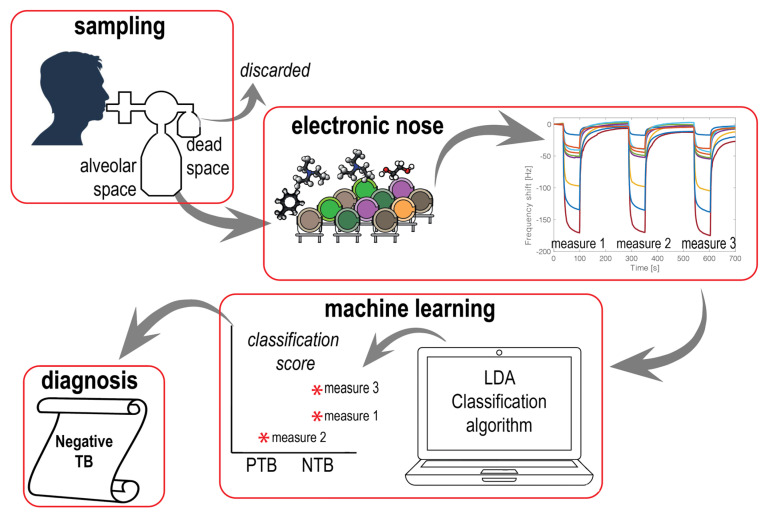
Schematic view of breath analysis. The content of the alveolar bag of the breath sampler is delivered to the electronic nose. Three short pulses of collected breath are sequentially measured. The three measures are processed by the linear discriminant analysis (LDA) classifier, each measure is assigned to a class, and the reliability of each assignment is evaluated with a classification score (marked with a star in the classification score plot). The final response of the diagnosis corresponds to the class predicted with the highest classification score.

PCA was calculated using standardized sensor data where the responses of each sensor were normalized to zero mean and unitary variance. Normalization is necessary to equalize the contribution of each sensor to the array. Indeed, the range of signals is different; an example of sensor signals is shown in Figure 2. The difference is due to several factors, such as the different sensitivity of sensors and the non-reproducibility of sensors’ manufacturing procedures.

It is important to consider that the principal components are calculated only considering the variance of the multivariate data. Such an unsupervised process defines directions where the total variance of the data is decomposed into uncorrelated variables. However, different directions may be defined to achieve an optimal separation between the classes. Linear discriminant analysis (LDA) enables the definition of variables (canonical variables), which are still obtained as a linear combination of sensors. With respect to PCA, LDA is a supervised algorithm that needs to be trained and tested. For this scope, the whole dataset was split into two subsets; one was used to train the model and the other to test it. LDA was calculated using standardized sensor data, and it was used to assign each measured sample to the TB or non-TB groups.

The whole data classification procedure is outlined in Figure 2. Three measurements for each subject were considered as independent, and the LDA model was assessed with this amount of data using 70% of the data to train the classifier and 30% of the data to test the model. Finally, each subject was assigned to the class that achieved the largest classification score. In LDA, the difference between the canonical variable for each sample and the mean of the class was considered as the score of the classifier. This quantity is used as an indication of the reliability of the class prediction. In practice, a sample measured three times was assigned to the class that corresponded to the largest classification score. This approach is similar to a majority voting-based classification where instead of comparing with many classifiers, more measurements from the same sample are compared.

In classification problems, the partition of data may strongly influence the performance of the algorithm. In particular, the random split of a dataset might result in either over-optimistic or over-pessimistic conclusions. To avoid this drawback, the LDA model was calculated 100 times. Each time, a different random partition of data was applied to train the model and to test it. In each iteration, a number of classification performance indicators were calculated. They are sensitivity, specificity, accuracy, and area under the ROC (AUROC). Finally, the LDA model achieving the average performance indicators was considered as the EN classification model.

Variance analysis, PCA, and machine learning classifications were performed in Matlab R2020b using the functions available in the Statistics and Machine Learning Toolbox (Mathworks, Natick, MA, USA).

## 3. Results and Discussion

In total, 100 breath samples were measured. There were 46 patients proven to be positive for pulmonary TB (PTB) with TB-LAMP and 38 healthy controls (HC). Additional groups of 10 non-PTB (NTB) subjects and 6 subjects with TB (PTBX) were measured to test the predictivity of EN on uncertain cases. NTB subjects had TB symptoms but negative TB-LAMP results, while in PTBX, in spite of negative TB-LAMP results, the TB was confirmed by symptoms, chest X-ray, and physician outcome. Table 2 shows the demographic and clinical data of all groups. All subjects of the PTB group presented cough symptoms (more than 2 weeks) and an average low BMI (19.5). Being underweight commonly has a synergistic effect on TB development [40]. Among the PTB group, 41.5% were patients who relapsed to PTB and who had been treated for 6 months with healing confirmation. All the recruited subjects were negative for antigenic COVID-19 tests.

### Sensor Data Evaluation

**Figure 3:**
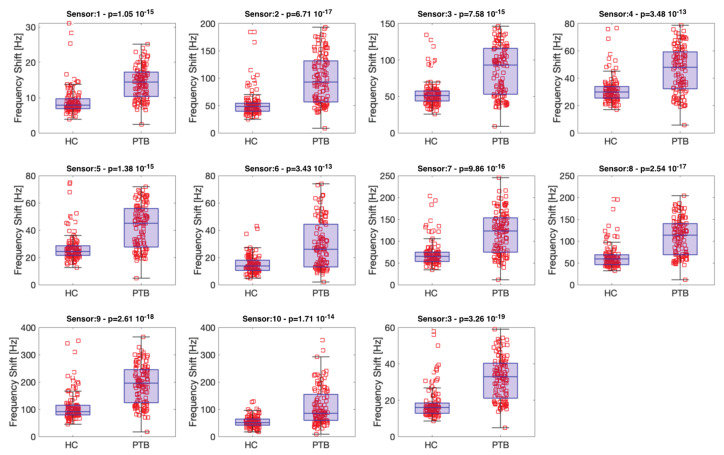
Kruskal–Wallis rank test and box and whisker plot of the responses of sensors to PTB and HC samples. Squares indicate the sensors measures, circles indicate the alleged outliers. All patients were measured in triplicate. Sensors are numbered according to the list in Table 1.

Figure 3 shows the distribution of the sensor signals in the two classes of PTB and HC. The statistical difference between the two groups is expressed by the *p*-value calculated with the Kruskal–Wallis nonparametric rank test. For all sensors, the mean of the frequency shift for the group of the PTB group is larger than that for the HC subjects. Despite the different formulation of sensitive materials, all sensors show a *p*-value lower than 0.001. Hence, for all sensors, the two classes are statistically different. Previous studies on VOCs released in the breath of TB patients indicated that the presence of TB is signaled by a specific pattern of a blend of aldehydes and methylated aromatics, alkenes, and alkanes [14], to which all sensors are expected, even if with a different intensity, to be sensitive [41].

Despite the low *p*-value, all sensors show an overlap of the two distributions. Thus, individual sensors are far from providing an ideal accuracy. However, sensors are elements of an array, and electronic noses have been introduced on the assumption that the collective behavior of the sensor array can overcome the limited capability of individual sensors to separate different groups. For the analysis of the sensor array data, the sensors’ responses to each sample are assembled to form vectors, and the signals collected from all samples are arranged in a matrix. The data matrix was analyzed with PCA and then with LDA.

**Figure 4:**
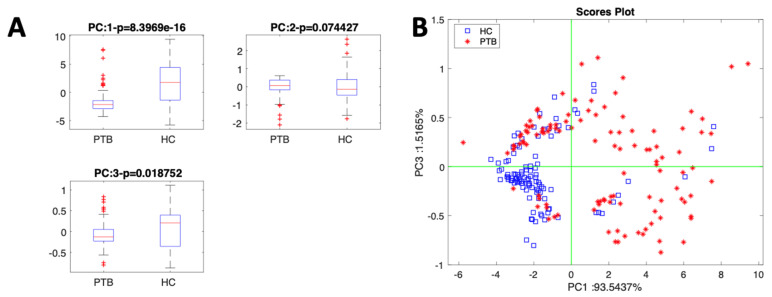
Results of the PCA of PTB and HC data. (**A**) Variance analysis of Kruskal–Wallis rank test. (**B**) Sensor data projected in the plane of the first and third principal component.

Figure 3 shows the main results of PCA. In Figure 4A, the *p*-values of the first three principal components with respect to the discrimination of the two groups are compared. PC1 and PC3 are the components where the two groups are better separated. In Figure 4B, the sensor data are projected in the plane defined by the first and the third principal component. In the plot, PC1 and PC3 account for more than 95% of the total variance of the sensor array, while PC2, for which a moderate class separation is obtained, explains 3% of the total variance. The other 2% of data variance is explained by higher-order principal components that do not contribute to the separation of classes. As expected from the boxplots in Figure 3, the two groups form distinct clusters with a limited overlap. Additionally, the group of PTB is characterized by a larger variability with respect to the controls. Indeed, even though all the positive TB subjects were enrolled before the TB treatment, their general physical states were rather different. For example, the range of weight loss is between 2 and 21 kg, and cough duration spanned from 2 to 36 weeks.

A more efficient evaluation of the performance of EN data in terms of capturing the differences between the classes can obtained using the supervised classification LDA algorithm. As detailed in the Materials and Methods section, LDA was calculated using the total dataset, where each subject was measured three times and then assigned to the class for which the largest classification score was obtained.

**Figure 5:**
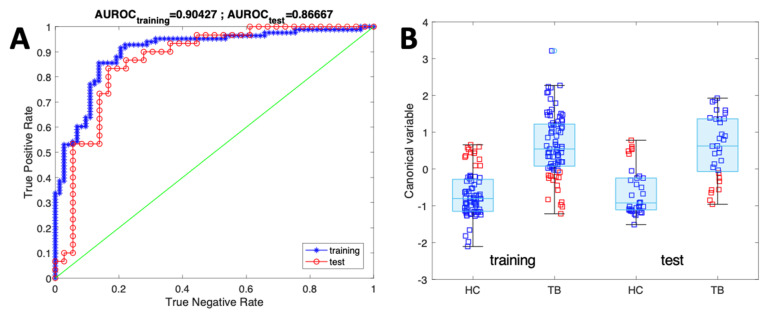
(**A**) ROC of the classifier of PTB with respect to HC in training and test. (**B**) Distribution of canonical variables in training and test. Blue and red squares indicate, respectively, the samples predicted correctly and incorrectly.

The performance of the classifier is shown in Table 3. For both training and test data, the accuracy, the sensitivity, the specificity, and the AUROC are listed. For each quantity, the interval of confidence at 95% was evaluated using the asymptotic formula. Figure 5 shows the ROC and the distribution of the canonical variables for the TB and HC groups in the training and test.

The model generated with the data collected from TB cases and healthy controls was applied to those patients who showed symptoms but had a negative TB-LAMP test. In total, 10 of these cases (labeled as NTB) were negative for TB, while in 6 cases (labeled as PTBX), the diagnosis of pulmonary tuberculosis was confirmed by further inspections based on chest X-rays and clinical condition evaluations. Table 4 shows the result of the model applied to these cases. The performance achieved by the classifier between PTB and HC is confirmed with the cases for which the current diagnostic tools provided contradictory results.

**Figure 6:**
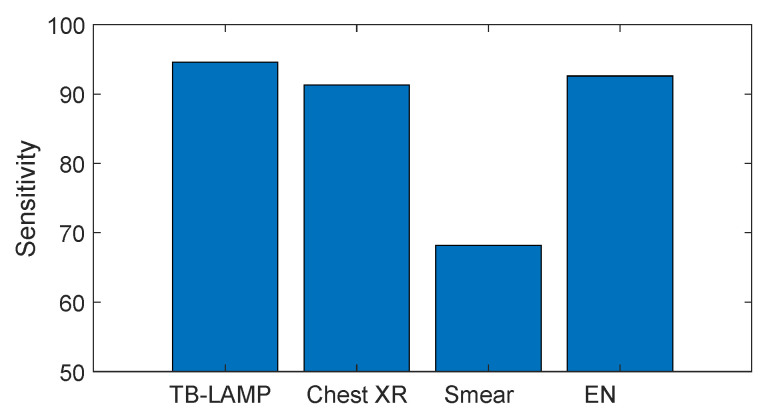
Comparison of the sensitivities shown by all diagnostic tools utilized during the present study.

Finally, in Figure 6, the sensitivities of all the methods used in this study are compared. It is interesting to note that the sensitivity of EN is comparable to TB-LAMP and chest-Xray, and largely outperforms SMEAR.

## 4. Conclusions

Previous studies suggest that electronic noses could be used to diagnose TB. In this paper, we exploited the potential of an EN in a clinical context, measuring patients during their routine interaction in a public hospital in Douala (Cameroon). The electronic nose was an advanced version of a device previously used to detect the change in breath composition of TB patients during therapy [28].

The results show that the tested EN can detect all positive TB patients with respect to healthy controls with an accuracy of 88.0% and a sensitivity and specificity of 90.8% and 85.7%, respectively. Furthermore, this performance is maintained in the prediction of cases for which the standard diagnostic tools provide a conflicting diagnosis.

In Table 5, the performance achieved in this study is compared with previous literature results from on-field tests. The results are substantially similar; in some cases, the effects of HIV on TB were considered, but inconsistent results were found [29,30]. It is important to remark that a lack of standardization of the studies and, moreover, the non-contemporaneous identification of the relevant VOCs with a standard analytical technique make is difficult to compare the results obtained with sensors based on different technologies.

Thus, the present results provide further evidence about the possibility of developing a fast, cheap, and portable POC device specifically devoted to TB diagnosis. Such a device would also give access to diagnosis and effective treatment to people living in remote areas, and would enable quick and correct detection of potentially infectious hospitalized patients. For this purpose, more studies are still necessary, not only to enforce the statistics, in particular with respect to comorbidities, but also to verify the robustness of the technology and the stability of the performance in very long-term operation.

## Figures and Tables

**Table 1:** List of the used sensitive molecules.

	Sensitive Molecule
1	5,10,15,20-tetrakis-(4-butyloxyphenyl)porphyrinCopper
2	5,10,15,20-tetrakis-(4-butyloxyphenyl)porphyrinCobalt
3	5,10,15,20-tetrakis-(4-butyloxyphenyl)porphyrinZinc
4	5,10,15,20-tetrakis-(4-butyloxyphenyl)porphyrinMagnesium
5	5,10,15,20-tetrakis-(4-butyloxyphenyl)porphyrinManganeseChloride
6	5,10,15,20-tetrakis-(4-butyloxyphenyl)porphyrin-IronChloride
7	5,10,15,20-tetrakis-(4-butyloxyphenyl)porphyrin-TinDichloride
8	2,3,7,8,12,13,17,18-octabromo-5,10,15,20-tetraphenylporphyrinH2
9	2,3,7,8,12,13,17,18-octabromo-5,10,15,20-tetraphenylporphyrin oxoMolybdenum
10	5,10,15-tris(3,5-dimethylphenyl)corroleCopper
11	5,10,15-tris(9-phenantryl)corrole

**Table 2:** Clinical and demographic data for PTP and HC patients.

Factor	PTB (#46 Subjects)	HC (38 Subjects)	PTBX (6 Subjects)	NTB (10 Subjects)
Sex (M/F)	30/16	23/15	4/2	6.82E-012/8
Age (mean, SD)	36.93 (13.96)	34.03 (9.90)	43.5(19.18)	40.3(10.87)
BMI (mean, SD)	19.511(2.86)	26.3364 (3.98)	23.68(5.62)	25.26(6.76)
VIH+	8	0	0	1
X-ray (positive)	39	N/A	6	0
SMEAR (positive)	18/30	N/A	0/6	0
C-RP (positive)	08/11	N/A	2/3	2/6
Fever	41	0	4	2
Cough (>2 weeks)	46	0	5	10
Appetite loss	45	0	0	0
Weight loss				
(2–21 kg)	33	0	6	6
Night sweats	40	0	1	0
Smokers	9	2	0	1
Alcohol drinkers	20	28	3	2
Recovered	9	N/A	1	0
TB history	17	N/A	0	3
Diabetic	1	0	0	1
MDR (Rifampicin)	1	N/A	N/A	0
Hypertension (HTA)	0	1	0	0

**Table 3:** Performance of the classification of PTB with respect to HC.

	Accuracy	Sensitivity	Specificity	AUROC
Training	89.8%	92.6 ± 10%	87.5 ± 11%	0.90
Test	88.0%	90.8 ± 17%	85.7 ± 18%	0.88

**Table 4:** Results of the application of the LDA classifier to the negative TB-LAMP of symptomatic patients.

	PTBX	NTB
Predicted as PTB	5	2
Predicted as HC	1	8

**Table 5:** Comparison of the achieved classification between PT and HC with previous literature studies.

Sensor Technology	Sensitivity	Specificity	Reference
Metal oxide semiconductors	93.5%	85.3%	[27]
Metal oxide semiconductors	88.0%	92.0%	[28]
Organic capped gold nanoparticles	85.0%	89.0%	[29]
Porphyrin-coated QMBs	94.0%	90.0%	[30]
Porphyrin- and corrole-coated QMBs	90.8%	85.7%	This work

## Data Availability

Not applicable.
